# In vivo gene expression in a *Staphylococcus aureus* prosthetic joint infection characterized by RNA sequencing and metabolomics: a pilot study

**DOI:** 10.1186/s12866-016-0695-6

**Published:** 2016-05-05

**Authors:** Yijuan Xu, Raluca Georgiana Maltesen, Lone Heimann Larsen, Henrik Carl Schønheyder, Vang Quy Le, Jeppe Lund Nielsen, Per Halkjær Nielsen, Trine Rolighed Thomsen, Kåre Lehmann Nielsen

**Affiliations:** Center for Microbial Communities, Department of Chemistry and Bioscience, Aalborg University, Fredrik Bajersvej 7H, 9220 Aalborg, Denmark; Department of Clinical Microbiology, Aalborg University Hospital, Aalborg, Denmark; Department of Clinical Medicine, Aalborg University Hospital, Aalborg, Denmark; The Danish Technological Institute, Life Science Division, Aarhus, Denmark; Section for Molecular Diagnostics, Department of Clinical Biochemistry, Aalborg University Hospital, Aalborg, Denmark

**Keywords:** *Staphylococcus aureus*, Joint infection, Prosthesis, In vivo gene expression, Virulence, Metabolism, Siderophore, RNA-seq, NMR, Metabolomics

## Abstract

**Background:**

*Staphylococcus aureus* gene expression has been sparsely studied in deep-sited infections in humans. Here, we characterized the staphylococcal transcriptome in vivo and the joint fluid metabolome in a prosthetic joint infection with an acute presentation using deep RNA sequencing and nuclear magnetic resonance spectroscopy, respectively. We compared our findings with the genome, transcriptome and metabolome of the *S. aureus* joint fluid isolate grown in vitro.

**Result:**

From the transcriptome analysis we found increased expression of siderophore synthesis genes and multiple known virulence genes. The regulatory pattern of catabolic pathway genes indicated that the bacterial infection was sustained on amino acids, glycans and nucleosides. Upregulation of fermentation genes and the presence of ethanol in joint fluid indicated severe oxygen limitation in vivo.

**Conclusion:**

This single case study highlights the capacity of combined transcriptome and metabolome analyses for elucidating the pathogenesis of prosthetic infections of major clinical importance.

**Electronic supplementary material:**

The online version of this article (doi:10.1186/s12866-016-0695-6) contains supplementary material, which is available to authorized users.

## Background

*Staphylococcus aureus* is one of the leading causes of community- and hospital-acquired infections worldwide. The clinical spectrum ranges from superficial skin lesions to deep-sited or generalized infections. Besides acute infections, *S. aureus* can adapt to a biofilm mode of growth in response to certain environmental cues and thereby infections become persistent and recurrent, particularly in association with prosthetic implants [1]. Moreover, the emergence and spread of resistance to many classes of antibiotics pose an increasing threat to public health. Consequently, staphylococci have been studied extensively both in vitro and in vivo with special focus on resistance and virulence. An arsenal of virulence factors has been identified including toxins, cell surfaces proteins that facilitate attachment and colonization, and factors that contribute to immune evasion and tissue damage [2]. However, few studies have investigated nutrient acquisition and metabolism of *S. aureus* in vivo during infection, which is an important aspect of *S. aureus* pathophysiology.

Recently, the increasing number of genome sequences of *S. aureus* have provided deeper insights into its virulence, antibiotic resistance and physiology in general [3]. It is recognized that the success of *S. aureus* depends not only on its virulence genes and development of antibiotic resistance, but also on a coordinated and timely expression of genes upon infection of its host. To elucidate this complicated orchestration of gene expression, the transcriptome has been studied in vitro and in vivo using rabbit [4] and mouse [5, 6] infection models. However, pathogens are likely to make host-specific adaptions by altering gene expression, which necessitates studies in humans. To our knowledge, Date et al. [6] is the only published investigation of the transcriptome of *S. aureus* in humans with cutaneous infections caused by the methicillin-resistant USA300 strain.

The aim of this study was to compare the in vivo expression of virulence and metabolic genes of *S. aureus* in a prosthetic joint infection in a human subject with growth in vitro as reference using RNA sequencing (RNA-seq). Moreover, using nuclear magnetic resonance (NMR) spectroscopy we analyzed the metabolites in the joint fluid and in culture supernatants in order to determine the biochemical composition of the environments.

## Results and discussion

### *S. aureus* infection: culture, genome and transcriptome

Standard culture of joint fluid, tissue biopsies, and prosthesis components revealed a pure growth of *S. aureus* with a pansusceptible antibiogram (see case history in Methods). Amplicon sequencing was used for detection of bacteria in fluid obtained by sonication of prosthesis components (all joint fluid was used for RNA-seq). Approximately 44000 reads were obtained, all of which were clustered into operational taxonomic units (OTUs) identified as *S. aureus* (data not shown).

The joint infection had an acute presentation although a previous indolent period cannot be precluded (see case history). Assuming an acute infection [7], we chose to compare gene expression of the in vivo sample with the isolate in an exponential growth phase. Additionally, we sequenced the genome of the isolate (SAU060112) to gain insight into the virulence and antibiotic resistance capacity and to facilitate high fidelity RNA-seq read mapping.

To reconstruct the genome 17.8 million reads were generated. The assembly resulted in 17 contigs with an average coverage of 729 and N50 of 601492 bp. The total length of contigs was 2.68 Mb which is close to the average (2.86 Mb) gapless chromosome length of *S. aureus* (currently 66 strains in total available at NCBI, May 2015). No plasmids were found. The genome assembly is predicted to contain 2562 protein-coding genes. Details of the assembly and analysis of the COG classification distribution of the protein-coding sequences can be found in Additional file 1: Tables S1, Additional file 2: Table S2 and Additional file 3: Figure S1. The isolate was spa type t908 and belonging to Clonal Complex 45. Interestingly, according to Driebe et al. [8] CC45 show less homoplasy density than other *S. aureus* clades indicating little recombination with other clonal complexes. Furthermore, in contrast to other CC45 strains included in the study, but similar to USA600-BAA1754 (spa type t671), the entererotoxin genes entC, sel and sen is present in SAU060112. We thus believe that within the CC45 complex, SAU060112 is relative closely related to USA600-BAA1754 despite the different spa type. Approximately 25 and 350 million RNA-seq reads were obtained for in vitro cultures and the in vivo sample, respectively (Table 1). Between 26.8 and 37.8 % of total reads from the in vitro cultures were mapped to the protein-coding sequences of the genome with the mapping criteria employed (95 % similarity, 80 % length fragment). Relaxation of the mapping criteria led to increased mapping efficiency (data not shown), however, this also increased the risk of erroneous mapping of human host transcripts to the bacterial genome. Thus, this conservative approach was chosen for all samples. As expected, the majority of the sequences from the in vivo sample originated from the human host, and only 1.2 % (4.1 million) reads were mapped to the *S. aureus* genome and 0.086 % (0.3 million) to the protein-coding sequences. While 0.3 million reads might be considered a relative low number of reads compared to modern RNA-Seq studies that frequently have many millions of reads per sample, it is still expected to be enough to detect reads from about 85 % of bacterial genes according to [9]. It is possible that other methods of purification of bacterial RNA from background host RNA than the one we employed can yield a higher proportion of bacterial RNA. A total of 430 genes (17 % of total) were found to be differentially expressed, of which 317 were upregulated and 113 downregulated in vivo*.* The complete list of differentially expressed genes is available in Additional file 4: Complete list of differentially expressed genes.

**Table 1:** Summary of RNA-seq mapping statistics (numbers of reads are in millions)

Sample	No of sequences	No of aligned reads (% of total sequence)	No of rRNA reads (% of aligned reads)	No of aligned mRNA reads (% of aligned reads)	R-value (biological replicates)
Joint fluid	348.4	4.1 (1.2)	3.8 (92.7)	0.3 (7.3)	-
LB culture					
1	26.7	18.1 (67.8)	8 (44.2)	10.1 (55.8)	>0.95
2	26.5	17.7 (66.8)	10.1 (57.1)	7.1 (40.1)	
3	23.1	15.7 (68.0)	7.8 (49.7)	7.5 (47.8)	

### Antibiotic resistance genes

SAU060112 was susceptible to β-lactams (including penicillin and methicillin) and 5 additional antibiotic classes. Analysis of the genome by the Resistance Gene Identifier (RGI) at the Comprehensive Antibiotic Research Database [10] predicted absence of resistance genes to β-lactams, macrolides and aminoglycosides in accordance with the antibiogram, but identified several efflux pumps related to other antibiotics (Additional file 5: Figure S2). Some of the efflux pumps (*tet38* 40-fold, p-val = 8.2*10^−40^; *mepA* 7-fold, p-val = 2.5*10^−5^) and cell wall biosynthesis genes (*mgt* 12-fold, p-val = 4.0*10^−10^; *pbp2* 5-fold, p-val = 0.0027; *murZ* 8-fold, p-val = 5.8*10^−6^) had increased expression in vivo*,* possibly induced by antibiotic treatment received by the patient for two days. The peptidoglycan biosynthesis pathway has been shown to be upregulated in *S. aureus* treated with subinhibitory doses of cell wall active antibiotics [11, 12]. However, several studies [12–14] have shown responses in bacteria is a global process not only involving proteins directly affected by antibiotics, but also proteins with no apparent relationship to the antibiotics. Therefore, it is unknown to which extent the differentially regulated genes found in this study was induced by antibiotic treatment or an in vivo response.

### Virulence

A total of 131 known or proposed virulence genes were found in the genome (Table 2, Additional file 6: Table S3), of which 47 were upregulated in vivo, including many toxins, several adhesins and immune evasion molecules. The highest upregulated toxin was γ-hemolysin (*hlgA* 776-fold, p-val = 1.6*10^−28^, *hlgB* 482-fold, p-val = 1.4*10^−29^, and *hlgC* 701-fold, p-val = 3.5*10^−31^), which has previously been found among the most overexpressed toxins in *S. aureus* cultivated in human blood in vitro [15] and in human cutaneous abscesses [6]. Among the in vivo upregulated extracellular matrix binding proteins, major histocompatibility complex (MHC) analogous protein (*map*) had the highest expression (458-fold, p-val = 2.7*10^−26^). *Map* was found significantly expressed in *S. aureus* during the acute phase of murine osteomyelitis [16] and the protein has been linked to severity of arthritis and osteomyelitis in this animal model [17].

**Table 2:** Differentially expressed virulence genes in vivo compared to in vitro. The RNA-seq data are compared with the microarray data of *Staphylococcus aureus* subsp. *aureus* USA300_FPR3757 (community-acquired methicillin-resistant) infected cutaneous abscesses in humans retrieved from Date et al. [6]

SAU060112	USA300	Gene name	Product	Fold change	Number/100000 mapped mRNA reads		Fold change during human cutaneous abscesses
					Infection	LB	
Toxins							
SAU060112_40253	SAUSA300_2365	hlgA	Gamma-hemolysin component A	776	574	1	12.56
SAU060112_40254	SAUSA300_2366	hlgC	Gamma-hemolysin component C	701	524	1	5.76
SAU060112_20343	SAUSA300_0396		Superantigen-like protein	503	78	0	1.48
SAU060112_40255	SAUSA300_2367	hlgB	Gamma-hemolysin component B	482	531	2	17.65
SAU060112_50039	SAUSA300_1974		Uncharacterized leukocidin-like protein 1	376	453	2	9.72
SAU060112_50038	SAUSA300_1975		Uncharacterized leukocidin-like protein 2	140	198	2	14.42
SAU060112_20344	SAUSA300_0398		Toxin, beta-grasp domain protein. superantigen-like protein	128	29	0	1.65
SAU060112_20345	-		Toxin, beta-grasp domain protein, superantigen-like protein	109	41	1	
SAU060112_20347	SAUSA300_0399	set	Exotoxin 3	104	23	0	1.1
SAU060112_10176	SAUSA300_1058	hly	Alpha-hemolysin	91	136	2	2.08
SAU060112_20348	SAUSA300_0401	set	Exotoxin 1	80	21	0	1.33
SAU060112_20350	SAUSA300_0403	ssl7nm	Enterotoxin-like toxin	56	8	0	1.34
SAU060112_110014	SAUSA300_1918		Truncated beta-hemolysin	36	2	0	7.84
SAU060112_10172	SAUSA300_1061		Superantigen-like protein	21	9	1	4.93
SAU060112_10173	SAUSA300_1060		Beta-grasp domain toxin protein, superantigen-like protein	17	9	1	2.64
SAU060112_10457	-	entC	Enterotoxin type C-2	15	72	7	
SAU060112_10174	SAUSA300_1059		putative superantigen-like protein	11	10	1	2.35
SAU060112_20351	SAUSA300_0404		Superantigen-like protein	9	6	1	1.39
SAU060112_10456	SAUSA300_0800	sel	Extracellular enterotoxin L	3	5	2	1.13
Exoenzymes							
SAU060112_20156	SAUSA300_0224	coa	Staphylocoagulase	12	55	7	1.5
SAU060112_40510	SAUSA300_2603	lip	Lipase 1	4	95	32	0.51
Adhesins							
SAU060112_110015	SAUSA300_1917	map	MHC analogous protein	458	2894	10	No data
SAU060112_10448	SAUSA300_0774	emp	Extracellular matrix protein-binding protein emp	77	50	1	2.02
SAU060112_40332	SAUSA300_2441	fnbA	Fibronectin-binding protein A	17	116	10	1.73
SAU060112_40330	SAUSA300_2440	fnbB	Fibronectin-binding protein B	5	108	31	1.67
SAU060112_10180	SAUSA300_1055	fib	Fibrinogen-binding protein	5	6	2	1.02
SAU060112_70131	SAUSA300_1327	ebh	Extracellular matrix-binding protein ebh	3	28	14	1.08
Immune evasion							
SAU060112_110009	SAUSA300_1920	chp	Chemotaxis inhibitory protein	29	11	1	6.1
SAU060112_10179	SAUSA300_1056	scn	Staphylococcal complement inhibitor	26	5	0	0.91
SAU060112_40252	SAUSA300_2364	sbi	Immunoglobulin-binding protein sbi	15	230	22	1.9
SAU060112_110010	SAUSA300_1919	scn	Staphylococcal complement inhibitor	3	27	15	1.55
SAU060112_20047	SAUSA300_0113	spa	Immunoglobulin G-binding protein A	−6	63	618	1.48
SAU060112_10182	SAUSA300_1053	flr	FPRL1 inhibitory protein	26	2	0	3.16
Exopolysaccharides							
SAU060112_20094	-	cap8J	Capsular polysaccharide synthesis enzyme Cap8J	−16	0	8	
Secretion system							
SAU060112_20221	SAUSA300_0285	esxB	Virulence factor EsxB	−10	1	382	0.85
SAU060112_20220	SAUSA300_0284	esaC	Protein EsaC	−9	1	9	0.83
SAU060112_20216	SAUSA300_0280	essA	Protein EssA	−9	1	9	1.3
SAU060112_20218	SAUSA300_0282	essB	Protein EssB	−8	1	12	0.86
SAU060112_20215	SAUSA300_0279	esaA	Protein EsaA	−6	6	56	0.61
SAU060112_20219	SAUSA300_0283	essC	Protein EssC	−5	10	81	0.79
Iron acquisition							
SAU060112_20052	SAUSA300_0118	sbnA	putative siderophore biosynthesis protein SbnA	27	14	1	7.02
SAU060112_20056	SAUSA300_0122	sbnE	IucA/IucC family siderophore biosynthesis protein	20	34	3	1.99
SAU060112_20055	SAUSA300_0121	sbnD	Transporter, major facilitator family protein	15	20	2	5.02
SAU060112_20057	SAUSA300_0123	sbnF	Siderophore biosynthesis protein	14	44	5	2.9
SAU060112_20053	SAUSA300_0119	sbnB	Ornithine cyclodeaminase	13	15	2	6.35
SAU060112_20054	SAUSA300_0120	sbnC	Siderophore biosynthesis protein, IucA/IucC family	11	24	3	5.17
SAU060112_20060	SAUSA300_0126	sbnI	conserved protein of unknown function	9	22	4	4.25
SAU060112_20059	SAUSA300_0125	sbnH	Conserved protein of unknown function	7	33	8	4.33
SAU060112_20058	SAUSA300_0124	sbnG	Conserved protein of unknown function	4	14	5	4
Virulence regulators							
SAU060112_110071	SAUSA300_1866	vraS	Sensor protein VraS	28	275	15	1.14
SAU060112_10560	SAUSA300_0691	saeR	Response regulator SaeR	19	309	24	3.72
SAU060112_110072	SAUSA300_1865	vraR	Response regulator protein VraR	14	141	15	0.63
SAU060112_10561	SAUSA300_0690	saeS	Histidine protein kinase SaeS	8	394	75	2.53
SAU060112_20048	SAUSA300_0114	sarS	HTH-type transcriptional regulator SarS	−13	1	18	0.35

The extraordinary ability of *S. aureus* to adapt to different physiological niches (e.g. the nares, skin, joints, blood, etc.) and cause a variety of clinical pictures is partly attributed to its many virulence determinants, which are tightly regulated and involve complex networks of regulatory factors [18]. Knowledge of its regulatory networks during colonization and infection in vivo remains limited due to the inherent complexity. Among the many virulence regulators, only *saeRS* (*saeR* 19-fold, p-val = 3.0*10^−10^; *saeS* 8-fold, p-val = 5.8*10^−6^) and *vraSR* (*vraS* 28-fold, p-val = 8.4*10^−13^; *vraR* 14-fold, p-val = 8.5*10^−11^) were highly induced in vivo (Table 2, Additional file 6: Table S3). Cell-wall-affecting antibiotics are known to induce *vraSR* and *saeRS* [19]. Thus, expression of both systems could be partly induced by β-lactams prior to surgery (see case history). *VraSR* positively regulates cell-wall peptidoglycan synthesis in *S. aureus* [20, 21]. *SaeRS* has a global impact on expression of virulence factors [22, 23] and is important for innate immune evasion by *S. aureus* [24]. Several virulence genes controlled by *saeRS* were highly upregulated, including *map,* α-(91-fold, p-val = 7.5*10^−27^), β- (36-fold, p-val = 2.1*10^−7^), γ-hemolysins (*hlgA* 776-fold, p-val = 1.6*10^−28^, *hlgB* 482-fold, p-val = 1.4*10^−29^, and *hlgC* 701-fold, p-val = 3.5*10^−31^), *chp* (29-fold, p-val = 9.2*10^−17^)*,* 2 loci for *scn* (26-fold, p-val = 3.9*10^−10^; 3-fold, p-val = 0.004)*,* coagulase (*coa*) (12-fold, p-val = 5.1*10^−14^), *sbi* (15-fold, p-val = 1.4*10^−9^*)*, extracellular matrix protein-binding protein (*emp*) (77-fold, p-val = 8.1*10^−36^), and two fibronectin binding proteins (17-fold, p-val = 3.7*10^−13^; 5-fold, p-val = 0.00011) [22, 23] (Table 2). *SaeRS* was also overexpressed in cutaneous abscesses in humans [6], murine osteomyelitis [16] as well as during incubation with human blood or serum [15].

Notably, the expression of 32 virulence genes present in the genome was negligible (≤5 reads/100,000 mapped mRNA reads) in vivo. Also, 9 virulence genes were found downregulated including transcription regulator *sarS* (13-fold, p-val = 6.1*10^−6^) [25], immunoglobulin G-binding protein A (*spa*) (6-fold, p-val = 0.005), and six of eight genes in the putative ESAT-6-secretion system, while expression of these genes was reported unchanged in [6] (Table 2). SarS belongs to the SarA protein family, global regulators of virulence gene expression in *S. aureus* [26]. SarS, which is controlled by many regulators, activates *spa* expression and represses α-hemolysin [18, 27]*.* This correlates with the finding in this study of expression of *spa* being reduced while expression of α-hemolysin is increased (91-fold) in vivo. ESAT-6 proteins have been reported to be important for staphylococcal infection in mice, but their functions during human infection remain unclear [28].

### Siderophores

In response to iron limitation, *S. aureus* has two known iron acquisition mechanisms: one is the iron-regulated surface determinant (*isd*) gene set that mediates heme acquisition from mammalian heme-containing proteins, and the other is a Fe(III)-siderophore acquisition system, which is capable of removing iron from human transferrin and lactoferrin. *S. aureus* produces two distinct siderophores: staphyloferrin A and staphyloferrin B [29]. The Ferric Uptake Regulator (Fur) controls expression of genes encoding all these systems [30], but mechanisms for fine-tuning of expression of these systems are unknown. We found 3-fold upregulation of *fur* (p-val = 0.004) during in vivo infection, but no difference in expression of *isd* and staphyloferrin A genes. However, the *sbn* operon (locus SAU060112_20052 – 20060) encoding staphyloferrin B was upregulated in vivo (3- to 27-fold, p-val = 6.9*10^−5^ – 2.3*10^−17^) in this study. The ninth protein SbnI encoded by the *sbn* operon is recently found to play an important role in transcription control of the *sbn* operon [31]. Staphyloferrin B production has been found to be important for *S. aureus* growth in iron-limited medium and for its pathogenicity in a murine kidney abscess model [32]. In human cutaneous abscesses expression of both *isd* and *sbn* operons was elevated as well as two genes of the staphyloferrin A operon [6].

### Metabolism

**Fig. 1:**
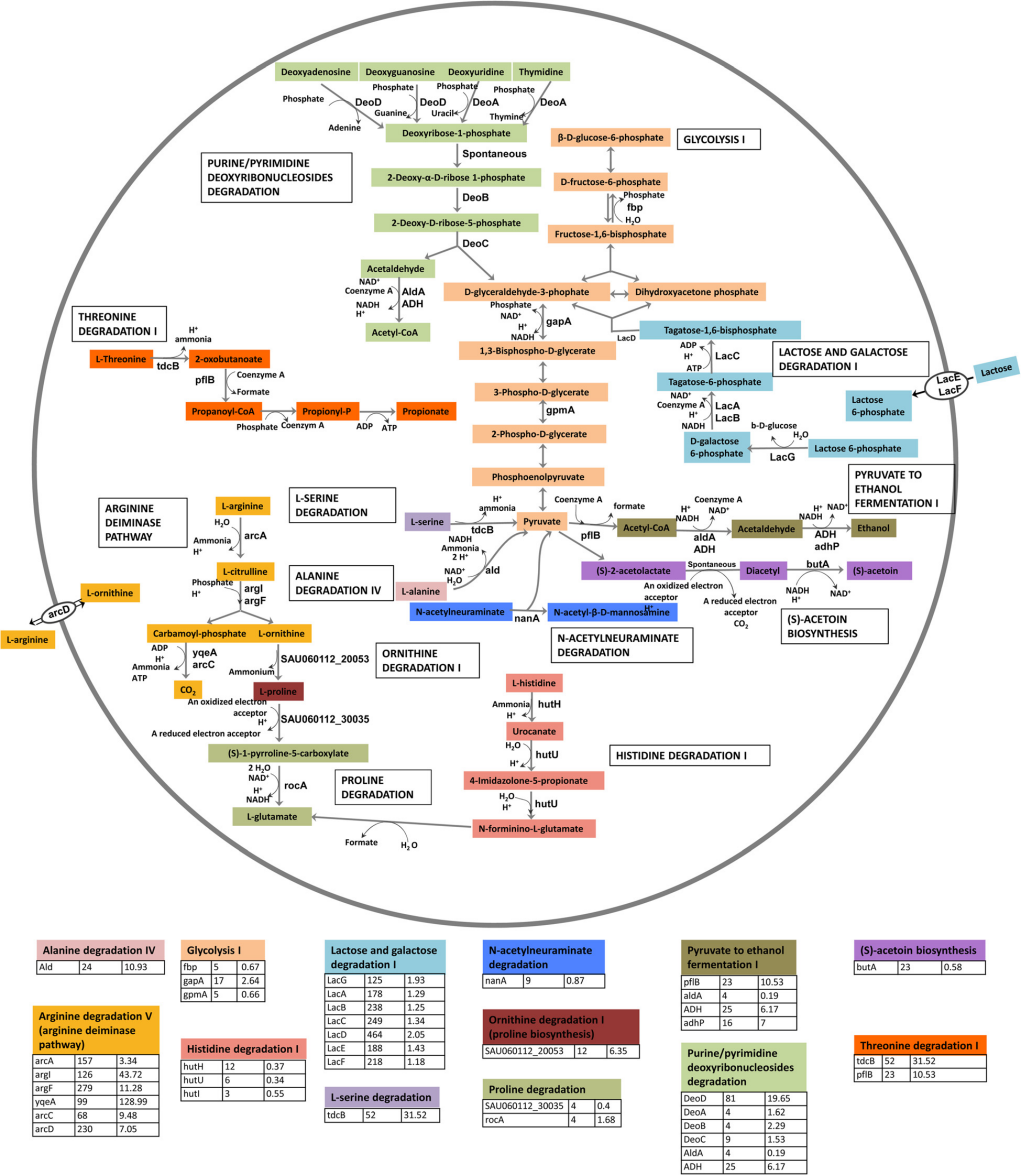
Overexpressed metabolic pathways in the infection. Pathway names are according to the MetaCyc database. Each pathway is assigned with a specific color and the upregulated enzymes in each pathway are indicated. On the bottom, under each pathway fold change of the upregulated enzymes in the current study are listed in the second column while fold change of these enzymes in the human cutaneous abscesses study [6] are in the third column

**Fig. 2:**
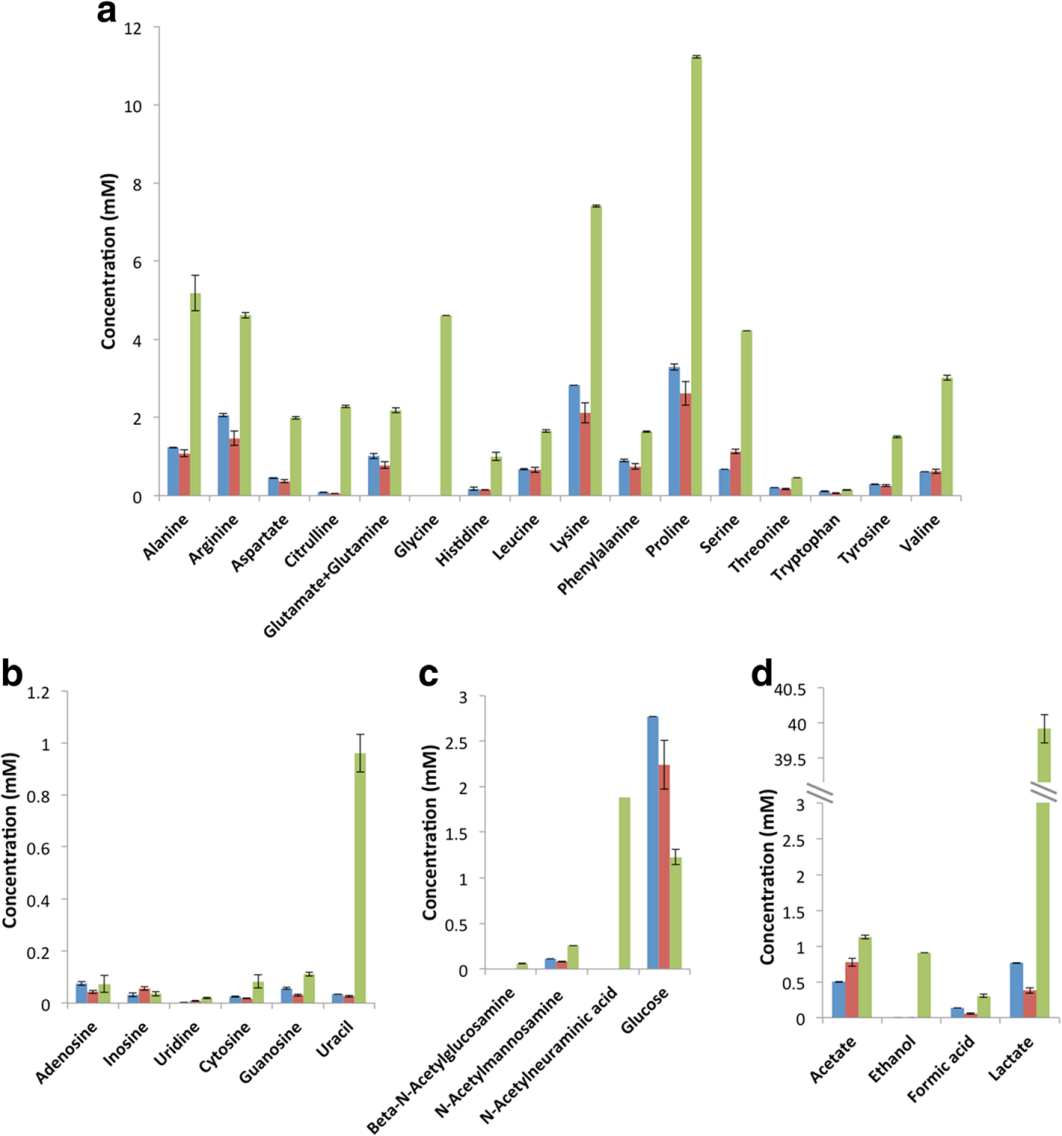
Concentration of metabolites determined by NMR analysis. In vitro (OD_600_ = 0) (*blue*) and joint fluid (*green*) were analyzed in technical triplicates while in vitro (OD_600_ = 0.5) (*red*) was done in biological replicates. The detection limit of NMR is ~ 2 μM. **a**: amino acids. **b**: nucleobases. **c**: glycans. **d**: metabolites

We observed upregulation of several genes related to anaerobic/hypoxic conditions, which include the genes involved in pyruvate to ethanol fermentation (*pflB* 23-fold, p-val = 8.3*10^−7^; *aldA* 4-fold, p-val = 7.9*10^−3^; *ADH* 25-fold, p-val = 2.9*10^−13^; *adhP* 16-fold, p-val = 1*10^−8^) and acetoin reductase (23-fold, p-val = 2.4*10^−10^) involved in pyruvate to acetoin fermentation as well as the upregulation of the arginine deiminase (ADI) pathway (*arcA* 156-fold, p-val = 1.0*10^−19^; *arcB* 279-fold, p-val = 4.3*10^−26^; *arcC* 67-fold, p-val = 3.5*10^−16^; *arcD*-230 fold, p-val = 1.2*10^−22^; *argI* 126-fold, p-val = 3.8*10^−35^) (Fig. 1) and pyruvate formate-lyase-activating enzyme (*pflA*) (63-fold, p-val = 7.3*10^−10^). The anaerobic/hypoxic condition was further supported by the high concentration of lactate (~40 mM) and presence of ethanol in the infected joint fluid (Fig. 2).

The ADI operon was the most upregulated amino acid catabolic pathway in the current study as well as in human cutaneous abscesses [6] and chronic human and murine osteomyelitis [16]. This operon also includes arginine/ornithine antiporter *arcD*, which is the only transporter for free arginine [33]. Arginine is utilized by *S. aureus* as a source of energy under anaerobic conditions [34]. We think that this pathway is essential for the direct production of ATP without generating organic acids under anaerobic conditions. This hypothesis is indirectly supported by the overexpression of the ethanol fermentation pathway. Under microaerophilic or anaerobic conditions, *S. aureus* ferments the majority of pyruvate to lactic acid in vitro [33]. However, lactic acid concentration was nearly 40 mM in the joint fluid (Fig. 2), which was higher than average lactate level in septic arthritides and probably was produced mainly by human host cells under hypoxic condition [35]. To avoid the unfavorable production of additional lactic acid while still oxidizing NADH to NAD^+^ for continuation of glycolysis and ATP generation, genes promoting pyruvate fermentation to ethanol were upregulated instead.

Besides ADI, high expression of catabolic threonine dehydratase *tdcB* (52-fold, p-val = 2.5*10^−19^), alanine dehydrogenase *ald* (24-fold, p-val = 1.3*10^−14^) and several additional amino acids catabolic enzymes were observed (Fig. 1), while several genes involved in amino acid synthesis including tryptophan, arginine, cysteine and histidine were among the 113 downregulated genes in vivo. Moreover, NMR data showed high concentration of free amino acids in the infected joint fluid compared to LB culture (Fig. 2). Taken together, our data suggest that free amino acids were a major source of carbon and energy for *S. aureus* in vivo.

Besides amino acids, several genes involved in carbohydrate catabolism had increased expression in vivo, including N-acetylneuraminate lyase *nanA* (9-fold, p-val = 8.9*10^−7^) and the *lac* operon (125- to 464-fold, p-val = 2.4*10^−43^-2.7*10^−23^). The enzyme NanA catalyzes the cleavage of N-acetylneuraminic acid (Neu5Ac), which is the predominant sialic acid in humans and is present as a terminal sugar on a wide range of glycoproteins and glycolipids. Host glycoproteins can be used as nutrient for bacteria [36], for example, *Streptococcus pneumoniae* can utilize human glycoconjugates as the sole source of carbon for growth [37]. The increased expression of *nanA* is consistent with the higher concentration of Neu5Ac in the joint fluid than the in vitro supernatant where it was undetectable (Fig. 2). The *S. aureus lac* operon is inducible by galactose and suppressed by glucose [38]. The concentration of galactose in vivo was at the baseline level in the NMR spectra, hence, it is unknown to which extent galactose is used as a nutrient.

The increased expression of purine and pyrimidine deoxyribonucleoside degradation pathways (*deoA* 5-fold, p-val = 0.0001; *deoB* 4-fold, p-val = 0.001; *deoC* 9-fold, p-val = 5.8*10^−8^ and *deoD* 81-fold, p-val = 8.3*10^−10^) indicated that the pathogen probably also acquired nucleosides as nutrients. The end products of these pathways are acetyl-CoA, a central metabolic intermediate, and D-glyceraldehyde-3-phosphate, an intermediate of glycolysis (Fig. 1). The metabolite measurement shows increased levels of nucleosides, particularly uracil, in vivo (Fig. 2). Uracil has been found elevated in joint fluid from rheumatoid arthritis patients [39]; however, the mechanism behind this is unknown.

Although the concentration of free amino acids, some glycans and nucleosides were higher in the joint fluid, the expression level of all hydrolytic exoenzymes but lipases remained low in vivo (Additional file 6: Table S3). This is in contrast to findings reported by Szafranska et al., who observed upregulation of many genes encoding secreted proteolytic enzymes in *S. aureus* during acute and chronic murine osteomyelitis [16]. A possible explanation for the low expression of hydrolytic exoenzymes in the current study is that hydrolysis of proteins and glycans might have been done by host enzymes as part of the inflammatory response. Neutrophils both release proteases themselves and activate proteases expressed by cells resident in tissues. Thus, the host response could provide *S. aureus* with the free amino acids, the glycans and other nutrients needed for growth in vivo.

Among the transport systems, oligopeptide permease (opp) transporters encoded by the opp-1 operon (locus SAU060112_40296 – 40300) were the most overexpressed transporter system (up to 101-fold, p-val = 2.2*10^−21^) along with the genes surrounding the operon (locus SAU060112_40295 – 40303). This operon was also highly overexpressed in cutaneous abscesses in humans [6]. The exact role of *opp-1* remains unknown, although it was found to impact in vivo growth of *S. aureus* in mouse and rabbit infection models [40].

A major limitation of our study is the lack of biological replicates, as we did not obtain other samples of *S. aureus* infected joint fluid during the study period. In an attempt to find similarities of *S. aureus* gene expression in infections in human subjects, thus corroborating the findings in independent experiments, we compared our RNA-seq data extensively with microarray data from *S. aureus* cutaneous abscesses in humans [6]. Although the two studies differed in type of infections, genetic background of *S. aureus* isolates, experimental setups and analytic methods, they had 113 upregulated and 13 downregulated genes in common, which correspond to 36 % upregulated and 12 % downregulated genes found in this study. The upregulated virulence genes included *saeRS*, a few toxins (particularly γ-hemolysin and two uncharacterized leukocidin-like proteins), and *chp* (Table 2). With regard to nutrient acquisition and metabolism, the elevated transcripts were those of the *sbn* operon, ADI operon, *tdcB*, *ald*, and several enzymes involved in nucleoside catabolism as well as ethanol fermentation (Fig. 1). Additionally, the opp-1 operon was overexpressed in both studies. The 13 downregulated genes in both studies included the virulence regulator *sarS* (13-fold, p-val = 6.1*10^−6^)*,* cystathionine γ-lyase (*mccB* 6-fold, p-val = 0.001, glyoxal reductase (*yvgN* 5-fold, p-val = 0.004), glycosyl-4,4′-diaponeurosporenoate acyltransferase (*crtO* 668-fold, p-val = 0.004), phosphoribosylformylglycinamidine synthase 1 (*purQ* 5 fold, p-val = 0.002), and a few conserved proteins of unknown function. All in all, the biological function and regulation of these up- and down- regulated genes need to be investigated by future in vivo studies.

## Conclusions

This single case study highlights the capacity of combined transcriptome and metabolome analyses for elucidating the pathogenesis of deep-sited infections with and without a foreign body. Future research should explore the in vivo physiology and virulence of *S. aureus*, which may ultimately lead to new strategies to combat *S. aureus* infections.

## Methods

### Case history

The patient was an adult male with a sero-negative polyarthritis since his youth. Debut of psoriasis led to a diagnosis of psoriatic arthritis after approximately two decades. He had undergone numerous surgical procedures and had joint implants in one hip, both knees, one elbow and one shoulder. Immunomodulatory therapy with adalimumab (Humira, Abbott US), a tumor necrosis factor (TNF)-α antibody , was started 26 months before the admission. The patient was admitted after a fall with subsequent swelling of the right knee. He was febrile (38.8 **°**C) and had marginal leukocytosis (12.0*10^9^/L) and highly elevated C-reactive protein (304 μg/mL, reference interval <10 μg/mL). A joint puncture revealed serous joint fluid (60 % mononuclear leukocytes) and 10^4^–10^5^ colony forming units of *S. aureus*, susceptible to penicillin, methicillin and 5 antibiotic classes other than β-lactam [41]. Intra-venous dicloxacillin was commenced on the 2nd day of admission, but changed to cefuroxim in combination with gentamicin due to spiking fever. *S. aureus* with the same antibiogram was obtained from blood culture and biopsies obtained during revision surgery with removal of the implant on the 4th day of admission. On the same day intravenous therapy was switched to penicillin G. The blood culture isolate was referred to Statens Serum Institut (Copenhagen, Denmark) for *spa*-typing as part of national surveillance (t908, annotated to Clonal Complex 45). Several months later the patient underwent surgical revision and removal of implants from the left elbow and the left hip. *S. aureus* infection with the same antibiogram was confirmed.

### Culture and antibiotic resistance test

Joint fluid, biopsies and prosthetic components were cultured according to [42] with an incubation period of 14 days (see Additional file 7). Species identification was done with a MALDI Biotyper CA System (Bruker Daltonics, Germany). Antimicrobial susceptibility testing was carried out as above [41]. The *S. aureus* isolate from prosthetic components was designated SAU060112.

### 16S rRNA gene amplicon sequencing and data analysis

DNA extraction was done using MolYsis complete5 (Molzym, Germany) according to the manufacturer’s instructions. For 16S rRNA amplicon sequencing, the V1-3 region was PCR amplified with bacterial primers 27 F and 534R in accordance with the protocol used by the Human Microbiome Project [43] and sequenced on a MiSeq DNA sequencer (Illumina, CA) [44]. The 16S rRNA amplicon data were analyzed using QIIME toolkit [45]. Raw sequences were demultiplexed and quality-filtered using the default parameters. Sequences were then clustered into OTUs based on 99 % sequence similarity and taxonomy assignment was done using the Greengenes database [46].

### Genome sequencing and annotation

*S. aureus* SAU060112 was grown overnight in LB medium. DNA was extracted using UltraClean® Microbial DNA Isolation kit (MO BIO Laboratories, Inc, CA) according to the manufacturer’s instructions. From 1 μg of DNA, a library for Illumina paired-end (PE) sequencing was constructed using NEBNext® Ultra^TM^ DNA Library Prep Kit for Illumina®(New England Biolabs, MA) according to the manufacturer’s instructions. Libraries were sequenced (2 × 150 bp) using Truseq SBS Kit v.3-HS Sequencing Kit (Illumina Inc.) on an Illumina HiSeq 2000 (Illumina Inc). Sequenced PE reads were imported into CLC genomics workbench v.6.5.1 (CLC Bio, Aarhus, Denmark) for assembly. Contigs were annotated using the web interface Magnifying Genomes (MaGe) of the MicroScope platform from GenoScope [47]. Automatic annotations provided by MaGe were curated manually to validate the presence or absence of genes of interest. Based on the annotations, the protein coding genes were classified into the Cluster of Orthologous Groups (COG) [48] functional categories using COG automatic classification tool at MaGe. Details of genome sequencing and annotation can be found in Additional file 7.

### RNA sample collection, extraction and sequencing

Immediately following aspiration the joint fluid was centrifuged at 12100 g for 2 min at room temperature and the pellet and supernatant were snap-frozen separately in liquid nitrogen. RNA from in vitro cultures (3 biological replicates) were isolated from cultures grown to exponential phase (OD600 ~ 0.5) in LB medium. The cell suspension was centrifuged and supernatant and pellet were snap-frozen separately. All samples were stored at −80 °C until RNA extraction or NMR analysis.

RNA was extracted using RiboPure™ Bacteria Kit (Ambion®, Life Technologies) except that the in vivo sample was homogenized in a mortar (precooled in liquid nitrogen) before RNA extraction. The RNA solutions were purified and concentrated using the MinElute PCR Purification Kit (Qiagen).

Twenty micrograms of in vivo-derived RNA was sequentially treated with the MICROB*Enrich*™ and MICROB*Express*™ kits (Ambion®) to deplete mammalian RNA and enrich bacterial mRNA, respectively. Four to six micrograms of in vitro-derived RNA was used. Sequencing libraries were prepared with the enriched microbial RNA using Illumina® TruSeq® RNA Sample Preparation Kit v2 according to the manufacturer’s instructions. Libraries were PE sequenced (2 × 150 bp) using Truseq SBS Kit v.3-HS Sequencing Kit on an Illumina HiSeq 2000.

### Differential gene expression analysis

Using the RNA-Seq analysis function in CLC Genomics Workbench, reads were aligned to the annotated SAU060112 genome allowing a minimum length fraction of 0.8 and minimum similarity fraction of 0.95. A table of read counts was used as input for differential gene expression analysis using edgeR using default settings [49]. Only genes with false discovery rate <0.05 using Benjamini and Hochberg’s algorithm [50] were classified as differentially expressed.

### NMR spectroscopy analysis

Prior to NMR measurements, samples were centrifuged at 4 °C for 5 min at 12100 g and kept on ice thereafter. Aliquots of 500 μL of supernatants were mixed with 100 μL 0.2 M phosphate buffer (pH 7.4, 99 % ^2^H_2_O, 0.3 mM DSA-d_6_ (4,4-dimethyl-4-silapentane-1-ammonium trifluoroacetate)). 600 μL of the mixture was transferred to a 5-mm NMR tube and analysis was performed immediately using a Bruker 600-MHz NMR spectrometer (Bruker BioSpin, Germany) equipped with a TCI (^1^H, ^13^C, ^15^N, and ^2^H lock) cryogenic probe operating at 600.13 MHz for ^1^H at 298.1 K. For the analysis, a T_2_ relaxation-edited Carr-Purcell-Meiboom-Gill (CPMG) [51] experiment was used (“cpmgpr1d” in Bruker library, spectral width 12019.23 Hz, time domain 65 K, relaxation delay 4 s, acquisition time 2.72 s, total spin-echo time 67.4 ms, 64 scans). Data was exponentially multiplied corresponding to a line broadening of 0.3 Hz, Fourier transformed, manually phase- and baseline- corrected, and calibrated to the chemical shift of the methyl signal of L-alanine at 1.48 ppm. Subsequently, spectra were overlapped and normalized to the reference peak of DSA-d_6_ at 0.01 ppm. Peaks showing differences in intensity were quantified using TopSpin v3.1 (Bruker BioSpin, Germany). For metabolite identification, we used an in-house metabolite database, Chenomx NMR library (suite 7.6), Human Metabolome Database [52], Madison Metabolomics Consortium Database from MetaboHunter [53], AMIX (v. 3.9.10, Bruker BioSpin), BRUKER BBIOREFCODE database (v. 2.7.0), and literature references [54, 55].

### Availability of supporting data

The annotated genome sequence data was submitted to the European Nucleotide Archive (accession nos. CCXN01000001-CCXN01000017). The RNA-seq data discussed in this publication have been deposited in NCBI’s Gene Expression Omnibus [56] and are accessible through GEO Series accession number GSE62091 (http://www.ncbi.nlm.nih.gov/geo/query/acc.cgi?acc=GSE62091).

### Ethics statement

This study was conducted within the framework of the ‘Prosthesis-Related Infection and Pain’ (PRIS) - Innovation project, a Danish multidisciplinary project. The ‘PRIS’ project was approved by the Regional Research Ethics Committee for North Denmark (N-20110022). The patient described in the study has given informed consent to participate in the study and the publication of data.

### Availability of data and materials

The sample that this case story is built upon does not exist anymore, since all have been used during this study. Raw data can be forwarded to interested parties by contacting the corresponding author.

## Additional files

Additional file 1: Table S1. Genome assembly details. (DOCX 12 kb) Additional file 2: Table S2. Details of the contigs from genome assembly. (DOCX 13 kb) Additional file 3: Figure S1. Clusters of Orthologous Groups (COG) classification distribution of the protein-coding genes and the number of up- and down- regulated genes in vivo in each category. (PPTX 55 kb) Additional file 4: Complete list of differentially expressed genes computed using EdgeR. FC: fold change; CPM: count per million; FDR: false discovery rate. (XLSX 84 kb) Additional file 5: Figure S2. Resistance wheel including the resistance genes predicted by the Resistance Gene Identifier (RGI) at the Comprehensive Antibiotic Research Database. (PPTX 425 kb) Additional file 6: Table S3. List of known and putative virulence genes in SAU060112. (DOCX 26 kb) Additional file 7: Supplemental methods. (DOCX 22 kb)

## Abbreviations

ADI: arginine deiminase
*coa*: coagulase
COG: Cluster of Orthologous Groups
*emp*: extracellular matrix protein-binding protein
Fur: Ferric Uptake Regulator
*isd*: iron-regulated surface determinant
MaGe: Magnifying Genomes
MHC: major histocompatibility complex
Neu5Ac: N-acetylneuraminic acid
NMR: nuclear magnetic resonance
opp: oligopeptide permease
OTUs: operational taxonomic units
*pflA*: pyruvate formate-lyase-activating enzyme
RGI: Resistance Gene Identifier
RNA-seq: RNA sequencing
*spa*: immunoglobulin G-binding protein A
