# Supplementary material for: In vivo gene expression in a Staphylococcus aureus prosthetic joint infection characterized by RNA sequencing and metabolomics: a pilot study
Source: BMC Microbiol. 2016 May 5;16:80. doi: 10.1186/s12866-016-0695-6 (PMC4858865; doi:10.1186/s12866-016-0695-6)
Supplement: Additional file 1: Table S1. — Genome assembly details. (DOCX 12 kb) [file 12866_2016_695_MOESM1_ESM.docx]

**Table S1** Genome assembly details.

|  | Number |
| --- | --- |
| Paired reads | 17.8 million |
| Total contig length | 2,676,915 bp |
| Contigs | 17 |
| N50 | 601,492 |
| Protein coding sequences | 2,562 |
| Protein coding density | 84.45% |
| tRNA | 57 |
| 16S rRNA | 8 |
| GC% | 32.8% |
