# Supplementary material for: In vivo gene expression in a Staphylococcus aureus prosthetic joint infection characterized by RNA sequencing and metabolomics: a pilot study
Source: BMC Microbiol. 2016 May 5;16:80. doi: 10.1186/s12866-016-0695-6 (PMC4858865; doi:10.1186/s12866-016-0695-6)
Supplement: Additional file 2: Table S2. — Details of the contigs from genome assembly. (DOCX 13 kb) [file 12866_2016_695_MOESM2_ESM.docx]

**Table S2** Details of the contigs from genome assembly. Contig 9, 13, and 15 have higher coverage than others. Contig 9 is 16S rRNA gene, contig 13 is 23S and 5S rRNA genes, while contig 15 is identified to contain transposase family protein and integrase core domain protein.

| Contig no. | Consensus length | Total read count | Single reads | Reads in pairs | Average coverage |
| --- | --- | --- | --- | --- | --- |
| 1 | 770265 | 3734653 | 316089 | 3418564 | 698 |
| 2 | 425597 | 2540329 | 219327 | 2321002 | 857 |
| 3 | 351932 | 1634303 | 138709 | 1495594 | 668 |
| 4 | 601492 | 3545046 | 303366 | 3241680 | 846 |
| 5 | 58180 | 300086 | 26114 | 273972 | 741 |
| 6 | 106564 | 577527 | 49891 | 527636 | 778 |
| 7 | 176694 | 783797 | 66747 | 717050 | 638 |
| 8 | 39290 | 229475 | 20661 | 208814 | 837 |
| 9 | 1515 | 66838 | 11438 | 55400 | 6119 |
| 10 | 43370 | 203305 | 18045 | 185260 | 671 |
| 11 | 72167 | 350250 | 29684 | 320566 | 699 |
| 12 | 15737 | 71668 | 6322 | 65346 | 656 |
| 13 | 2980 | 135428 | 18378 | 117050 | 6289 |
| 14 | 3610 | 20262 | 2220 | 18042 | 803 |
| 15 | 1293 | 42557 | 6869 | 35688 | 4759 |
| 16 | 3487 | 14725 | 1591 | 13134 | 608 |
| 17 | 1141 | 5598 | 962 | 4636 | 709 |
