# Supplementary material for: In vivo gene expression in a Staphylococcus aureus prosthetic joint infection characterized by RNA sequencing and metabolomics: a pilot study
Source: BMC Microbiol. 2016 May 5;16:80. doi: 10.1186/s12866-016-0695-6 (PMC4858865; doi:10.1186/s12866-016-0695-6)
Supplement: Additional file 3: Figure S1. — Clusters of Orthologous Groups (COG) classification distribution of the protein-coding genes and the number of up- and down- regulated genes in vivo in each category. (PPTX 55 kb) [file 12866_2016_695_MOESM3_ESM.pptx]

## Slide 1
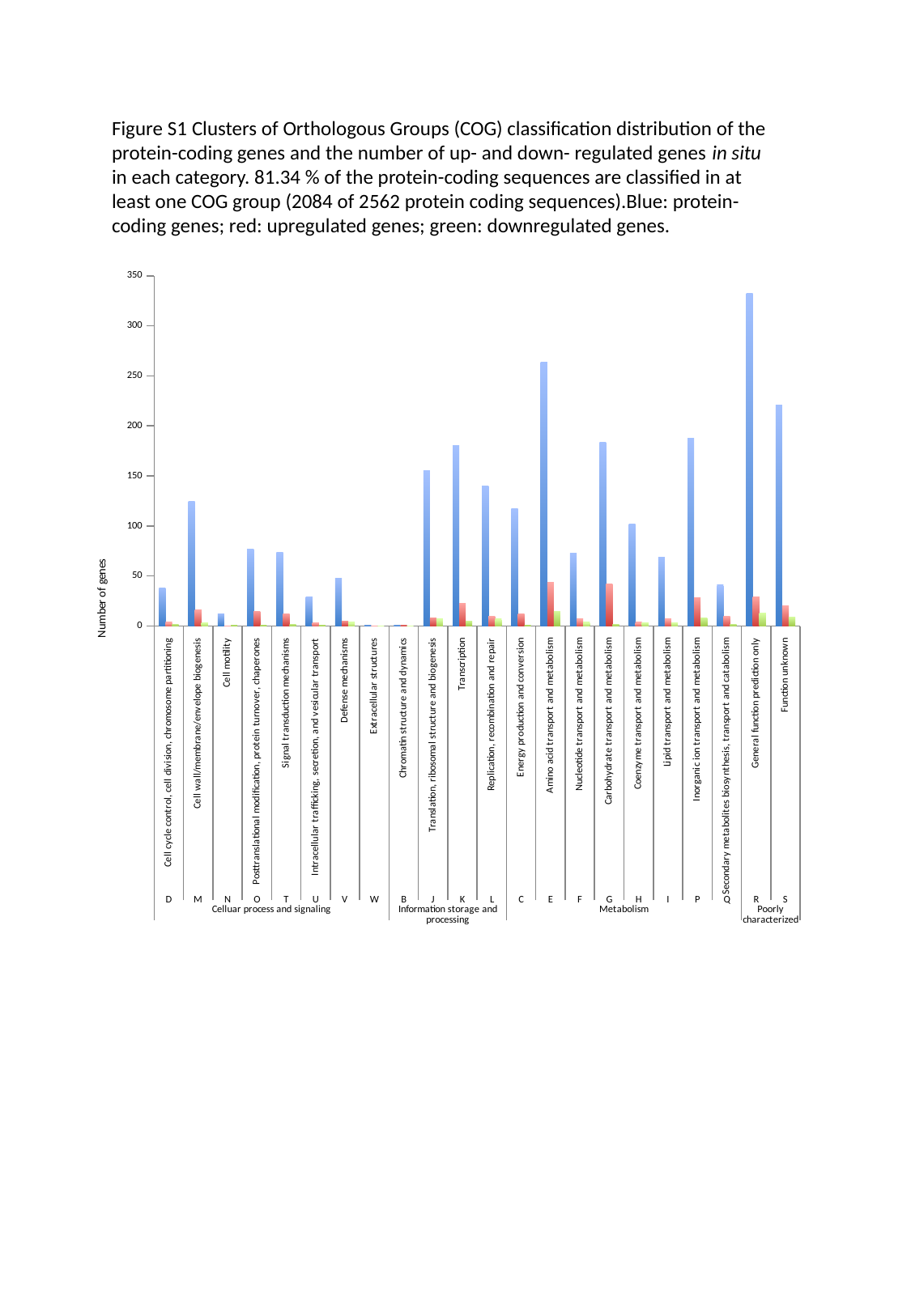

Figure S1 Clusters of Orthologous Groups (COG) classification distribution of the protein-coding genes and the number of up- and down- regulated genes in situ in each category. 81.34 % of the protein-coding sequences are classified in at least one COG group (2084 of 2562 protein coding sequences).Blue: protein-coding genes; red: upregulated genes; green: downregulated genes.
### Chart
| Category | Proteins | Up | Down |
|---|---|---|---|
| Cell cycle control, cell division, chromosome partitioning | 38.0 | 4.0 | 2.0 |
| Cell wall/membrane/envelope biogenesis | 125.0 | 16.0 | 3.0 |
| Cell motility | 12.0 | 0.0 | 1.0 |
| Posttranslational modification, protein turnover, chaperones | 77.0 | 15.0 | 1.0 |
| Signal transduction mechanisms | 74.0 | 12.0 | 2.0 |
| Intracellular trafficking, secretion, and vesicular transport | 29.0 | 3.0 | 1.0 |
| Defense mechanisms | 48.0 | 5.0 | 4.0 |
| Extracellular structures | 1.0 | 0.0 | 0.0 |
| Chromatin structure and dynamics | 1.0 | 1.0 | 0.0 |
| Translation, ribosomal structure and biogenesis | 155.0 | 8.0 | 7.0 |
| Transcription | 180.0 | 23.0 | 5.0 |
| Replication, recombination and repair | 140.0 | 10.0 | 7.0 |
| Energy production and conversion | 117.0 | 12.0 | 1.0 |
| Amino acid transport and metabolism | 264.0 | 44.0 | 15.0 |
| Nucleotide transport and metabolism | 73.0 | 7.0 | 4.0 |
| Carbohydrate transport and metabolism | 184.0 | 42.0 | 2.0 |
| Coenzyme transport and metabolism | 102.0 | 4.0 | 3.0 |
| Lipid transport and metabolism | 69.0 | 7.0 | 3.0 |
| Inorganic ion transport and metabolism | 188.0 | 28.0 | 8.0 |
| Secondary metabolites biosynthesis, transport and catabolism | 41.0 | 10.0 | 2.0 |
| General function prediction only | 332.0 | 29.0 | 13.0 |
| Function unknown | 221.0 | 20.0 | 9.0 |
