# Supplementary material for: In vivo gene expression in a Staphylococcus aureus prosthetic joint infection characterized by RNA sequencing and metabolomics: a pilot study
Source: BMC Microbiol. 2016 May 5;16:80. doi: 10.1186/s12866-016-0695-6 (PMC4858865; doi:10.1186/s12866-016-0695-6)
Supplement: Additional file 5: Figure S2. — Resistance wheel including the resistance genes predicted by the Resistance Gene Identifier (RGI) at the Comprehensive Antibiotic Research Database. (PPTX 425 kb) [file 12866_2016_695_MOESM5_ESM.pptx]

## Slide 1
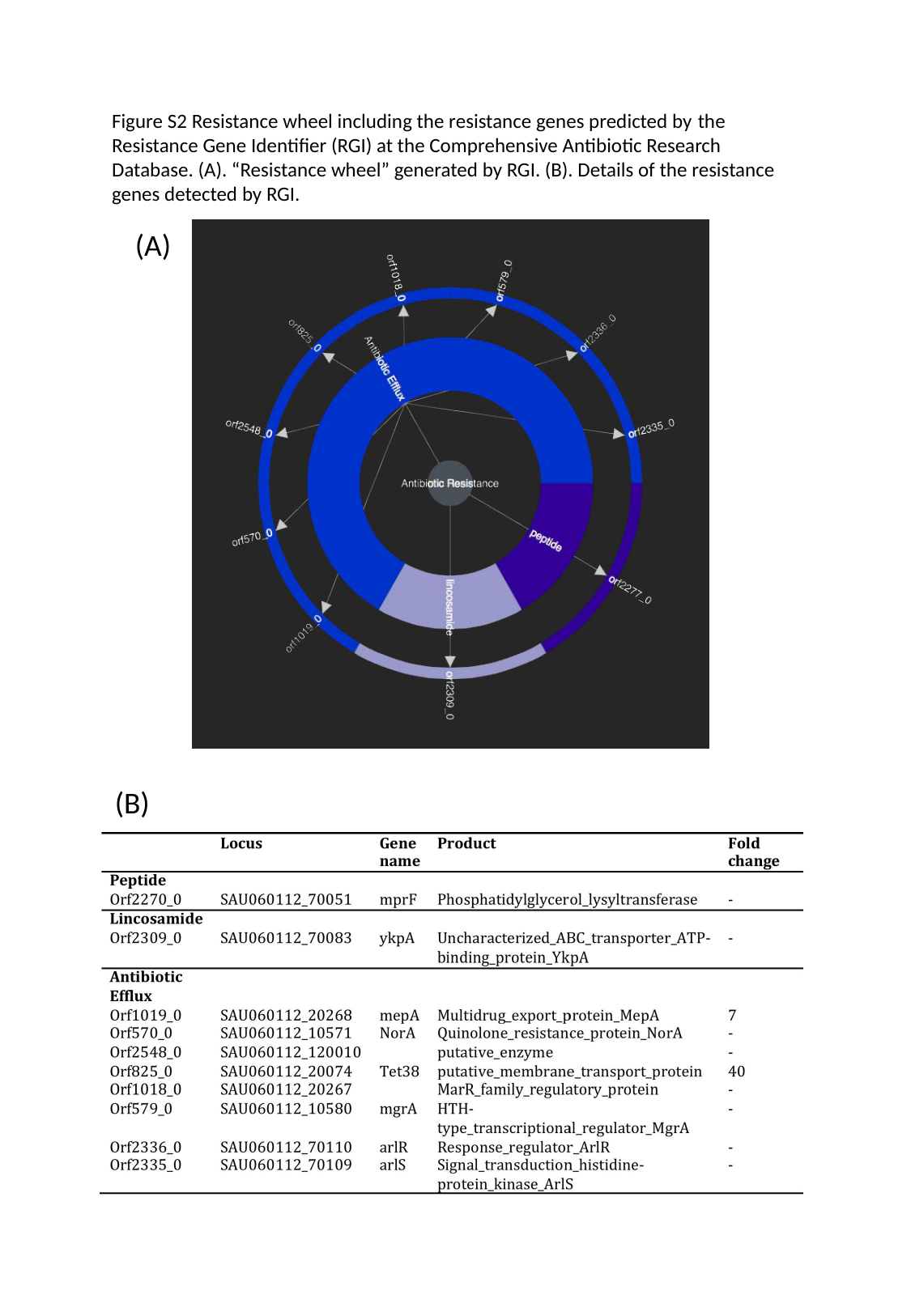

Figure S2 Resistance wheel including the resistance genes predicted by the Resistance Gene Identifier (RGI) at the Comprehensive Antibiotic Research Database. (A). “Resistance wheel” generated by RGI. (B). Details of the resistance genes detected by RGI.
(A)
(B)
