# Supplementary material for: In vivo gene expression in a Staphylococcus aureus prosthetic joint infection characterized by RNA sequencing and metabolomics: a pilot study
Source: BMC Microbiol. 2016 May 5;16:80. doi: 10.1186/s12866-016-0695-6 (PMC4858865; doi:10.1186/s12866-016-0695-6)
Supplement: Additional file 6: Table S3. — List of known and putative virulence genes in SAU060112. (DOCX 26 kb) [file 12866_2016_695_MOESM6_ESM.docx]

**Table S3** List of known and putative virulence genes in SAU060112. In the second column homologs in reference genome *S. aureus* subsp*. aureus* N315 (SA) are listed. If no homolog is found in *S. aureus* subsp*. aureus* N315, homologs in *S. aureus* subsp. *aureus* MW2 (MW) or *S. aureus* subsp. *aureus* Newman (NWMN) are given.

|  |  |  |  |  | **No/100,000 mapped mRNA reads** | |
| --- | --- | --- | --- | --- | --- | --- |
| **SAU060112** | **Reference genome** | **Gene name** | **Product** | **Fold change** | **Infection** | **LB** |
| **Exoenzymes** |  |  |  |  |  |  |
| SAU060112_20156 | SA0222 | coa | Staphylocoagulase | 12 | 55 | 7 |
| SAU060112_40510 | SA2463 | lip | Lipase 1 | 4 | 95 | 32 |
| SAU060112_20255 | SA0309 | lip | Lipase 2 |  | 222 | 181 |
| SAU060112_10288 | SA0900 | sspB | Staphopain B |  | 2 | 6 |
| SAU060112_10287 | SA0901 | sspA | Glutamyl endopeptidase |  | 4 | 14 |
| SAU060112_40040 | SA2003 | hysA | Hyaluronate lyase |  | 10 | 7 |
| SAU060112_40471 | SA2430 | aur | Zinc metalloproteinase aureolysin |  | 5 | 31 |
| **Plasminogen activator** | |  |  |  |  |  |
| SAU060112_110007 | SA1758 | sak | Staphylokinase |  | 4 | 4 |
| **Toxins** |  |  |  |  |  |  |
| SAU060112_40253 | SA2207 | hlgA | Gamma-hemolysin component A | 776 | 574 | 1 |
| SAU060112_40254 | SA2208 | hlgC | Gamma-hemolysin component C | 701 | 524 | 1 |
| SAU060112_20343 | SA0383 |  | Superantigen-like protein | 503 | 78 | 0 |
| SAU060112_40255 | SA2209 | hlgB | Gamma-hemolysin component B | 482 | 531 | 2 |
| SAU060112_50039 | SA1812 |  | Uncharacterized leukocidin-like protein 1 | 376 | 453 | 2 |
| SAU060112_50038 | SA1813 |  | Uncharacterized leukocidin-like protein 2 | 140 | 198 | 2 |
| SAU060112_20344 | SA0385 |  | Toxin, beta-grasp domain protein | 128 | 29 | 0 |
| SAU060112_20345 | SA0384 |  | Toxin, beta-grasp domain protein | 109 | 41 | 1 |
| SAU060112_20347 | SA0386 | set | Exotoxin 3 | 104 | 23 | 0 |
| SAU060112_10176 | SA1007 | hly | Alpha-hemolysin | 91 | 136 | 2 |
| SAU060112_20348 | SA0387 | set | Exotoxin 1 | 80 | 21 | 0 |
| SAU060112_20350 | SA0389 | ssl7nm | Enterotoxin-like toxin | 56 | 8 | 0 |
| SAU060112_110014 | SA1752 |  | Truncated beta-hemolysin | 36 | 2 | 0 |
| SAU060112_10172 | SA1011 |  | Superantigen-like protein | 21 | 9 | 1 |
| SAU060112_20306 | SA0357 |  | conserved protein of unknown function | 17 | 3 | 0 |
| SAU060112_10173 | SA1010 |  | Beta-grasp domain toxin protein | 17 | 9 | 1 |
| SAU060112_10457 | SA1817 | entC | Enterotoxin type C-2 | 15 | 72 | 7 |
| SAU060112_10174 | SA1009 |  | putative superantigen-like protein | 11 | 10 | 1 |
| SAU060112_20351 | SA0390 |  | Superantigen-like protein | 9 | 6 | 1 |
| SAU060112_10456 | SA1816 | sel | Extracellular enterotoxin L | 3 | 5 | 2 |
| SAU060112_10165 | NWMN_1082 | eta | exfoliative toxin A |  | 7 | 15 |
| SAU060112_50029 | SAS065 |  | Delta-hemolysin |  | 13 | 21 |
| SAU060112_20342 | SA0382 |  | Superantigen-like protein 11 |  | 16 | 10 |
| SAU060112_20354 | SA0393 |  | Superantigen-like protein 11 |  | 2 | 1 |
| SAU060112_30192 | SA1430 | entA | Enterotoxin type A |  | 1 | 2 |
| SAU060112_100039 | SA1642 | entG | Enterotoxin type G |  | 3 | 2 |
| SAU060112_100038 | SA1643 | sen | Enterotoxin SeN |  | 0 | 4 |
| SAU060112_100037 | SA1644 | entC | Enterotoxin type C-3 |  | 0 | 4 |
| SAU060112_100036 | SA1646 |  | Enterotoxin type I |  | 1 | 3 |
| SAU060112_100035 | SA1647 |  | Enterotoxin type I |  | 0 | 2 |
| SAU060112_100034 | SA1648 | seo | Staphylococcal enterotoxin O |  | 1 | 5 |
| absent | MW1378 | LukF | Panton-Valentine leukocidin chain F precursor |  |  |  |
| absent | MW1379 | LukS | Panton-Valentine leukocidin chain S precursor |  |  |  |
| absent | SA1819 | tst | toxic shock syndrome toxin-1 |  |  |  |
| **Adhesins** |  |  |  |  |  |  |
| SAU060112_110015 | SA1750 | map | MHC analogous protein | 458 | 2894 | 10 |
| SAU060112_10448 | SA0744 | emp | Extracellular matrix protein-binding protein emp | 77 | 50 | 1 |
| SAU060112_40332 | SA2291 | fnbA | Fibronectin-binding protein A | 17 | 116 | 10 |
| SAU060112_40330 | SA2290 | fnbB | Fibronectin-binding protein A | 5 | 108 | 31 |
| SAU060112_10180 | SA1003 | fib | Fibrinogen-binding protein | 5 | 6 | 2 |
| SAU060112_70131 | SA1267 | ebh | Extracellular matrix-binding protein ebh | 3 | 28 | 14 |
| SAU060112_30309 | SA1312 | ebpS | Elastin-binding protein EbpS |  | 71 | 202 |
| SAU060112_40505 | SA2458 | icaR | Biofilm operon icaADBC HTH-type negative transcriptional regulator IcaR |  | 2 | 6 |
| SAU060112_40506 | SA2459 | icaA | Poly-beta-1,6-N-acetyl-D-glucosamine synthase |  | 0 | 0 |
| SAU060112_40507 | SA2460 | icaD | Poly-beta-1,6-N-acetyl-D-glucosamine synthesis protein IcaD |  | 0 | 0 |
| SAU060112_40508 | SA2461 | icaB | Poly-beta-1,6-N-acetyl-D-glucosamine N-deacetylase |  | 0 | 1 |
| SAU060112_40509 | SA2462 | icaC | putative poly-beta-1,6-N-acetyl-D-glucosamine export protein |  | 0 | 1 |
| SAU060112_40528 | MW2612 | cna | Collagen adhesin |  | 21 | 125 |
| SAU060112_10450 | SA0742 | clfA | Clumping factor A |  | 200 | 158 |
| SAU060112_40464 | SA2423 | clfB | Clumping factor B |  | 87 | 107 |
| SAU060112_10708 | SA0519 | sdrC | Serine-aspartate repeat-containing protein C |  | 4 | 5 |
| SAU060112_10707 | SA0520 | sdrD | Serine-aspartate repeat-containing protein D |  | 16 | 68 |
| SAU060112_10706 | SA0521 | sdrE | Serine-aspartate repeat-containing protein E |  | 97 | 787 |
| **Exopolysaccharides** | |  |  |  |  |  |
| SAU060112_20094 | MW1033 | cap8J | Capsular polysaccharide synthesis enzyme Cap8J | -16 | 0 | 8 |
| SAU060112_20085 | MW1024 | cap8A | Capsular polysaccharide type 8 biosynthesis protein cap8A |  | 2 | 4 |
| SAU060112_20086 | MW1025 | capB | putative tyrosine-protein kinase CapB |  | 3 | 9 |
| SAU060112_20087 | MW1026 | capC | putative tyrosine-protein phosphatase CapC |  | 3 | 6 |
| SAU060112_20088 | MW1027 | capD | Capsular polysaccharide biosynthesis protein CapD |  | 8 | 19 |
| SAU060112_20089 | MW1028 | capD | UDP-glucose 4-epimerase |  | 4 | 11 |
| SAU060112_20090 | MW1029 | cap8F | Cap8F |  | 6 | 10 |
| SAU060112_20091 | MW1030 | capG | UDP-N-acetylglucosamine 2-epimerase |  | 4 | 8 |
| SAU060112_20092 | MW1031 | cap8H | Cap8H |  | 3 | 5 |
| SAU060112_20093 | MW1032 |  | putative membrane protein |  | 2 | 7 |
| SAU060112_20095 | MW1034 |  | Polysaccharide biosynthesis protein |  | 1 | 3 |
| SAU060112_20096 | MW1035 |  | Capsular polysaccharide synthesis enzyme Cap8L |  | 6 | 7 |
| SAU060112_20097 | MW1036 |  | putative UDP-galactose phosphate transferase (WeeH) |  | 3 | 10 |
| SAU060112_20098 | MW1037 |  | Polysaccharide biosynthesis family protein |  | 1 | 3 |
| SAU060112_20099 | MW1038 | rffD | UDP-N-acetyl-D-mannosaminuronic acid dehydrogenase |  | 7 | 14 |
| SAU060112_20100 | MW1039 | rffE | UDP-N-acetyl glucosamine-2-epimerase |  | 6 | 16 |
| **Secretion system** |  |  |  |  |  |  |
| SAU060112_20221 | SA0278 | esxB | Virulence factor EsxB | -10 | 1 | 382 |
| SAU060112_20220 | SA0277 | esaC | Protein EsaC | -9 | 1 | 9 |
| SAU060112_20216 | SA0273 | essA | Protein EssA | -9 | 1 | 9 |
| SAU060112_20218 | SA0275 | essB | Protein EssB | -8 | 1 | 12 |
| SAU060112_20215 | SA0272 | esaA | Protein EsaA | -6 | 6 | 56 |
| SAU060112_20219 | SA0276 | essC | Protein EssC | -5 | 10 | 81 |
| SAU060112_20214 | SA0271 | esxA | Virulence factor EsxA |  | 11 | 68 |
| SAU060112_20217 | SA0274 | esaB | Protein EsaB |  | 0 | 2 |
| **Iron acquisition** |  |  |  |  |  |  |
| **isd genes** |  |  |  |  |  |  |
| SAU060112_10209 | SA0976 | isdB | Iron-regulated surface determinant protein B |  | 3 | 1 |
| SAU060112_10208 | SA0977 | isdA | Iron-regulated surface determinant protein A |  | 7 | 15 |
| SAU060112_10207 | SA0978 | isdC | Iron-regulated surface determinant protein C |  | 0 | 1 |
| SAU060112_10206 | SA0979 | isdD |  |  | 2 | 2 |
| SAU060112_10205 | SA0980 | isdE | High-affinity heme uptake system protein IsdE |  | 1 | 1 |
| SAU060112_10204 | SA0981 | isdF | putative heme-iron transport system permease protein IsdF |  | 0 | 3 |
| SAU060112_10202 | SA0982 | srtB |  |  | 0 | 1 |
| SAU060112_10201 | SA0983 | isdG | Heme-degrading monooxygenase IsdG |  | 0 | 1 |
| **sbnABCDEFGHI** |  |  |  |  |  |  |
| SAU060112_20052 | SA0112 | sbnA | putative siderophore biosynthesis protein SbnA | 27 | 14 | 1 |
| SAU060112_20056 | SA0116 | sbnE | IucA/IucC family siderophore biosynthesis protein | 20 | 34 | 3 |
| SAU060112_20055 | SA0115 | sbnD | Transporter, major facilitator family protein | 15 | 20 | 2 |
| SAU060112_20057 | SA0117 | sbnF | Siderophore biosynthesis protein | 14 | 44 | 5 |
| SAU060112_20053 | SA0113 | sbnB | Ornithine cyclodeaminase | 13 | 15 | 2 |
| SAU060112_20054 | SA0114 | sbnC | Siderophore biosynthesis protein, IucA/IucC family | 11 | 24 | 3 |
| SAU060112_20060 | SA0120 | sbnI | conserved protein of unknown function | 9 | 22 | 4 |
| SAU060112_20059 | SA0119 | sbnH | Conserved protein of unknown function | 7 | 33 | 8 |
| SAU060112_20058 | SA0118 | sbnG | Conserved protein of unknown function | 4 | 14 | 5 |
| **Immune evasion** |  |  |  |  |  |  |
| SAU060112_110009 | SA1755 | chp | Chemotaxis inhibitory protein | 29 | 11 | 1 |
| SAU060112_10179 | SA1004 | scn | Staphylococcal complement inhibitor | 26 | 5 | 0 |
| SAU060112_40252 | SA2206 | sbi | Immunoglobulin-binding protein sbi | 15 | 230 | 22 |
| SAU060112_110010 | SA1754 | scn | Staphylococcal complement inhibitor | 3 | 27 | 15 |
| SAU060112_20047 | SA0107 | spa | Immunoglobulin G-binding protein A | -6 | 63 | 618 |
| SAU060112_10182 |  | flr | FPRL1 inhibitory protein | 26 | 2 | 0 |
| **Virulence regulators** |  |  |  |  |  |  |
| SAU060112_110071 | SA1701 | vraS | Sensor protein VraS | 28 | 275 | 15 |
| SAU060112_10560 | SA0661 | saeR | Response regulator SaeR | 19 | 309 | 24 |
| SAU060112_110072 | SA1700 | vraR | Response regulator protein VraR | 14 | 141 | 15 |
| SAU060112_10561 | SA0660 | saeS | Histidine protein kinase SaeS | 8 | 394 | 75 |
| SAU060112_20048 | SA0108 | sarS | HTH-type transcriptional regulator SarS | -13 | 1 | 18 |
| SAU060112_10649 | SA0573 | sarA | Transcriptional regulator SarA |  | 6 | 8 |
| SAU060112_40102 | SA2062 | sarV | HTH-type transcriptional regulator SarV |  | 1 | 1 |
| SAU060112_10598 | SA0623 | sarX | HTH-type transcriptional regulator SarX |  | 1 | 1 |
| SAU060112_40223 | SA2174 | sarZ | HTH-type transcriptional regulator SarZ |  | 10 | 21 |
| SAU060112_30037 | SA1583 | rot | HTH-type transcriptional regulator rot |  | 1 | 2 |
| SAU060112_10580 | SA0641 | MgrA | HTH-type transcriptional regulator MgrA |  | 22 | 30 |
| SAU060112_50025 | SA1844 | agrA | Accessory gene regulator protein A |  | 88 | 177 |
| SAU060112_50028 | SA1842 | agrB | Accessory gene regulator protein B |  | 66 | 206 |
| SAU060112_50027 | SAS066 | agrD | Accessory gene regulator protein D |  | 7 | 13 |
| SAU060112_50026 | SA1843 | agrC | Accessory gene regulator protein C |  | 109 | 427 |
| SAU060112_40131 | SA2089 | sarR | HTH-type transcriptional regulator SarR |  | 3 | 2 |
| SAU060112_30300 | SA1323 | srrA | Transcriptional regulatory protein SrrA |  | 45 | 68 |
| SAU060112_30301 | SA1322 | srrB | Sensor protein SrrB |  | 68 | 103 |
| SAU060112_10608 | SA0614 | graR | Response regulator protein GraR |  | 4 | 7 |
| SAU060112_10607 | SA0615 | graS | Sensor histidine kinase GraS |  | 7 | 12 |
| SAU060112_70109 | SA1246 | arlS | Signal transduction histidine-protein kinase ArlS |  | 13 | 13 |
| SAU060112_70110 | SA1248 | arlR | Response regulator ArlR |  | 6 | 6 |
| SAU060112_10338 | SA0856 | spxa | Regulatory protein spx |  | 102 | 160 |
| SAU060112_70093 | SA1233 | Msa | Protein msa |  | 0 | 0 |
| SAU060112_60021 | SA1886 | murF | UDP-N-acetylmuramoyl-tripeptide--D-alanyl-D-alanine ligase |  | 62 | 72 |
| **Others** |  |  |  |  |  |  |
| SAU060112_40399 | SA2353 | ssaA | Staphylococcal secretory antigen ssaA1 |  | 2 | 12 |
| SAU060112_40135 | SA2093 | ssaA | Staphylococcal secretory antigen ssaA2 |  | 27 | 71 |
