# Supplementary material for: In vivo gene expression in a Staphylococcus aureus prosthetic joint infection characterized by RNA sequencing and metabolomics: a pilot study
Source: BMC Microbiol. 2016 May 5;16:80. doi: 10.1186/s12866-016-0695-6 (PMC4858865; doi:10.1186/s12866-016-0695-6)
Supplement: Additional file 7: — Supplemental methods. (DOCX 22 kb) [file 12866_2016_695_MOESM7_ESM.docx]

**Supplemental methods**

**Sonication of prosthesis and culture**

The removed prosthesis components were first covered with 1x phosphate buffered saline (DNA/RNA free) and vortexed for 30 seconds followed by sonication at 40 kHz for 5 minutes to dislodge attached bacteria and a final vortex for 30 seconds. 50 ml of the sonication fluid was then centrifuged and the pellet was resuspended in 1ml of 1x PBS buffer and cultured as described for joint fluid. Sonication and centrifugation was done at room temperature.

**DNA extraction and sequencing**

The *S. aureus* isolate obtained by culture was grown overnight in Luria-Bertani (LB) medium. DNA was extracted using UltraClean^®^ Microbial DNA Isolation kit (MO BIO Laboratories, Inc) according to manufacturer’s instructions. DNA concentration was measured with the dsDNA BR Assay Kit on a Qubit 2.0 Fluorometer and its integrity verified using Agilent 2200 TapeStation system. From 1 μg of DNA, a library for Illumina paired-end (PE) sequencing was constructed using NEBNext^®^ Ultra^TM^ DNA Library Prep Kit for Illumina^®^(New England Biolabs) according to manufacturer’s instructions. Libraries were paired end (PE) sequenced (2 x 150 bp) using Truseq SBS Kit v.3-HS Sequencing Kit (Illumina Inc.) on an Illumina HiSeq 2000 (Illumina Inc).

**Genome assembly and annotation**

Sequenced PE reads were imported into CLC genomics workbench v.6.5.1 (CLC Bio, Aarhus, Denmark) and quality trimmed. Reads shorter than 50 bases after trimming or reads containing ambiguous nucleotides were discarded. Adapters sequences were removed if found. Trimmed PE reads were assembled using CLC’s *de novo* assembly algorithm with a kmer of 63 and a minimum scaffold size of 1000. The assembly was evaluated by mapping PE reads to the assembled scaffolds using CLC’s map reads back to contigs function at 95% similarity and with length fraction of 100%. The contigs were annotated using the web interface Magnifying Genomes (MaGe) of the MicroScope platform from GenoScope (1–3). The automatic annotations provided by MaGe were curated manually to validate the presence or absence of genes of interest. Based on the annotations, the protein coding genes were classified into the Cluster of Orthologous Groups (4) functional categories using COG automatic classification tool at MaGe.

1. Vallenet D, Labarre L, Rouy Z, Barbe V, Bocs S, Cruveiller S, et al. MaGe: a microbial genome annotation system supported by synteny results. Nucleic Acids Res. 2006; 34(1):53–65.

2. Vallenet D, Engelen S, Mornico D, Cruveiller S, Fleury L, Lajus A, et al. MicroScope: a platform for microbial genome annotation and comparative genomics. Database. 2009; 2009(0):bap021–bap021.

3. Vallenet D, Belda E, Calteau A, Cruveiller S, Engelen S, Lajus A, et al. MicroScope--an integrated microbial resource for the curation and comparative analysis of genomic and metabolic data. Nucleic Acids Res. 2013; 41(Database issue):D636–647.

4. Tatusov RL, Koonin EV, Lipman DJ. A genomic perspective on protein families. Science. 1997; 278(5338):631–637.
